# Association of blood trace minerals and nocturia in young and middle-aged adults: a cross-sectional study based on NHANES

**DOI:** 10.3389/fnut.2025.1545089

**Published:** 2025-06-25

**Authors:** Quanxin Su, Yanyu Zhang, Weiwei Fan, Qizhen Tang, Kenan Wang

**Affiliations:** ^1^Department of Urology, The First Affiliated Hospital of Dalian Medical University, Dalian, Liaoning, China; ^2^Department of Hematology, The First Affiliated Hospital of Dalian Medical University, Dalian, Liaoning, China

**Keywords:** trace minerals, selenium, nocturia, young and middle-aged, NHANES

## Abstract

**Objective:**

The mechanism underlying nocturia in young and middle-aged individuals remains unclear. This study aimed to investigate the association between blood levels of trace minerals and the occurrence of nocturia.

**Methods:**

This cross-sectional study utilized data from the 2021–2023 National Health and Nutrition Examination Survey (NHANES). Linear regression models and subgroup analyses were employed to assess the relationship between blood concentrations of trace minerals including lead, cadmium, total mercury, manganese, and selenium and nocturia. Dose–response relationships were analyzed using smoothed curve fitting.

**Results:**

A total of 2,099 participants were included in the analysis. In unadjusted regression models, blood levels of cadmium and manganese were significantly and positively associated with nocturia in young adults, whereas blood selenium levels were significantly and inversely associated. These associations remained statistically significant after adjusting for relevant covariates in multivariate linear regression analyses. Subgroup analysis revealed that among participants who reported alcohol consumption, those with higher blood cadmium levels had a significantly increased risk of nocturia (OR = 1.626, 95% CI: 1.305–2.026, *p* < 0.0001). Interaction testing indicated a significant difference in the effect of blood cadmium on nocturia across drinking status. A threshold effect was observed for selenium: when blood selenium levels were below 2.15 μmol/L, the risk of nocturia decreased significantly as selenium levels increased (*p* < 0.05). No significant associations were found between blood levels of lead or mercury and nocturia in any of the models.

**Conclusion:**

Blood levels of cadmium, manganese, and selenium are significantly associated with nocturia in young and middle-aged adults. These findings suggest that urologists should consider the potential role of trace mineral levels in the prevention and management of nocturia.

## 1 Introduction

Nocturia is a common lower urinary tract symptom, defined by the International Continence Society (ICS) in 2002 as “the complaint that the individual has to wake at night one or more times to void” (1). Following the standardization of this definition, numerous studies have explored the prevalence of nocturia and its associated risk factors. A survey conducted in the United States involving 5,024 community-dwelling individuals, with a mean age of 45.8 years, reported that 31% experienced nocturia, with 14.2% experiencing two or more episodes per night (2). In a meta-analysis of 42 studies, Bosch et al. found that the prevalence of nocturia (defined as ≥2 episodes per night) among older men aged 70 years or above ranged from 29 to 59.3%, while among older women it ranged from 28.3 to 61.5% (3). A substantial body of evidence indicates that nocturia is strongly associated with a variety of adverse outcomes, including fatigue, daytime drowsiness, reduced productivity and quality of life, cognitive impairment, obesity, diabetes mellitus, depression, and cardiovascular disease (2, 4). High-quality meta-analyses have also shown that nocturia is linked to an approximately 20% increased risk of falls, a 32% increased risk of fractures, and a 1.3-fold higher risk of mortality among the elderly (5, 6). Although nocturia is relatively uncommon in younger populations, its frequency increases significantly with age (7, 8). In individuals aged 60 years and older, the reported prevalence ranges from 30 to 80% (9). However, in recent years, there has been a noticeable rise in the proportion of younger individuals experiencing nocturia. The pathophysiological mechanisms underlying nocturia in younger adults are thought to be more complex and less well understood. As a result, the effective prevention and management of nocturia in younger patients poses a growing challenge for urologists.

In recent years, increasing attention has been directed toward the intake and physiological levels of trace minerals and their impact on chronic diseases, including their potential role in urinary function. This shift in focus is largely driven by global environmental changes and evolving dietary patterns. Trace minerals are widely present in both the environment and food sources. Among them, selenium and manganese are essential nutrients selenium is known for its antioxidant properties (10, 11), while manganese plays a key role in protecting cells from oxidative stress (12, 13). Conversely, lead, cadmium, and mercury are considered potentially toxic trace elements and may exert detrimental effects on urinary health.

It is hypothesized that managing trace mineral levels may offer a novel approach to addressing nocturia. This cross-sectional study, utilizing data from the National Health and Nutrition Examination Survey (NHANES), aims to explore the association between blood levels of five trace minerals selenium, manganese, lead, cadmium, and mercury and the occurrence of nocturia in adults under the age of 60. Additionally, the study investigates potential dose–response relationships between these trace minerals and nocturia, as well as whether these associations differ across specific population subgroups. The findings are expected to provide new insights into the prevention and management of nocturia among young and middle-aged adults.

## 2 Materials and methods

### 2.1 Study population

The National Health and Nutrition Examination Survey (NHANES) is an ongoing program designed to assess the health and nutritional status of the United States population. It utilizes a complex, multistage, probability sampling design to ensure a nationally representative sample. The survey collects comprehensive data on a wide range of health and nutrition indicators, as well as associated behaviors. Detailed information about the continuous design and methodology of NHANES is available at http://www.cdc.gov/nchs/nhanes/index.htm. All survey protocols were reviewed and approved by the Ethics Review Board of the National Center for Health Statistics, and informed consent was obtained from all participants prior to data collection.

**Figure 1:**
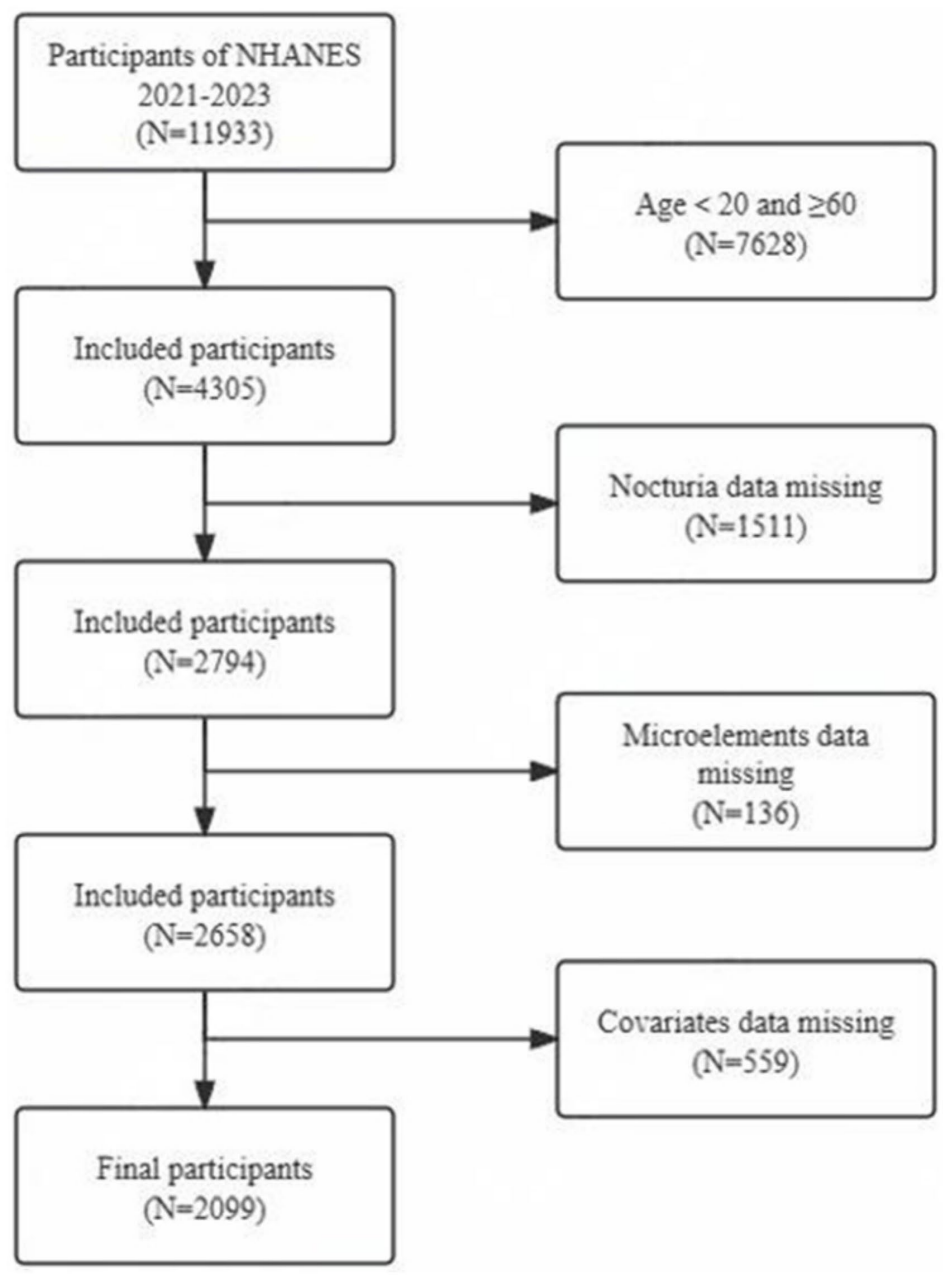
Flowchart of participant selection.

For this cross-sectional analysis, we extracted the most recent data available from NHANES, covering the period from August 2021 to August 2023. To avoid potential bias due to the impact of the COVID-19 pandemic, data from this cycle were not pooled with those from previous cycles. The participant selection process was carefully designed to ensure the representativeness and validity of the study sample. From the initial pool of 11,933 eligible individuals, we excluded 7,628 participants who were either younger than 20 or older than 60 years, 1,511 participants with missing nocturia data, 136 participants with missing blood trace element data, and 559 participants with incomplete covariate information. This resulted in a final analytical sample of 2,099 individuals. Figure 1 presents a flowchart detailing the participant selection process.

### 2.2 Assessment of nocturia

Nocturia in this study was assessed using the following survey question: *“In the past 30 days, during a typical night, how many times did you wake up to urinate?”* Participants who reported experiencing two or more nocturnal voids per night were classified as having nocturia.

### 2.3 Classification of blood trace elements

In this study, blood trace elements were treated as the independent variables. Whole blood samples were collected, processed, stored, and transported to the National Center for Environmental Health at the Centers for Disease Control and Prevention (CDC) in Atlanta, Georgia, for analysis. The concentrations of lead (Pb), cadmium (Cd), total mercury (Hg), manganese (Mn), and selenium (Se) were measured directly from whole blood specimens using mass spectrometry following a standardized dilution-based sample preparation protocol.

### 2.4 Covariate collection and definitions

In this study, a series of covariates associated with nocturia were synthesized, falling into two broad categories: demographic indicators and health status. The demographic indicators included age, gender, race, marital status, educational level, and poverty rate. Poverty rates were calculated based on monthly household income relative to the federal poverty level and were subsequently categorized as follows: 1 (low income) and 1–5 or >5 (middle/high income). The health status of the subjects was determined by several criteria, including alcohol consumption (defined as fewer than 12 drinks in the past year as “no” and 12 or more drinks as “yes”), smoking status (based on a history of smoking more than 100 cigarettes), body mass index (BMI), hypertension (defined as a previous diagnosis of high blood pressure or an average systolic blood pressure >140 mmHg and/or diastolic blood pressure >90 mmHg), hyperlipidemia (defined as a previous diagnosis of hyperlipidemia or the use of lipid-lowering medication), and diabetes (defined as a previous diagnosis of diabetes or the use of glucose-lowering medication).

### 2.5 Statistical analysis

The histogram was used to assess whether continuous variables conformed to a normal distribution. Continuous variables that followed a normal distribution were expressed as mean ± standard deviation (SD), while those that did not were expressed as median (first quartile, third quartile). Differences between participants grouped by nocturia status were assessed using weighted t-tests for continuous variables and weighted chi-square tests for categorical variables. Three logistic regression models univariate and multivariate were constructed to analyze the association between blood trace mineral levels and nocturia. Model 1 was unadjusted. Model 2 was adjusted for demographic variables, including age, sex, race, marital status, income-to-poverty ratio, and education level. Model 3 was further adjusted for health-related variables, including alcohol consumption, smoking status, BMI, hypertension, hyperlipidemia, and diabetes. Subgroup analyses were subsequently performed to assess the consistency of findings across demographic and health-related subgroups, thereby enhancing the generalizability of the results. The relationship and potential threshold effects between blood micronutrient levels and nocturia were examined using fitted smoothing curves and a threshold effect analysis model. The significance of the inflection point was assessed using a likelihood ratio test.

All statistical analyses were conducted using R Studio (version 4.2.2) and EmpowerStats (version 2.0). A *p*-value of less than 0.05 was considered statistically significant. A weighting strategy was applied to minimize the impact of substantial fluctuations within the dataset.

## 3 Results

### 3.1 Baseline characteristics of participants

A total of 2,099 study participants were included in this analysis. Table 1 presents the weighted sociodemographic characteristics of the participants, stratified by nocturia status. The mean age of the participants was 40.88 ± 11.39 years, with 44.69% of the participants being male, 58.41% non-Hispanic White, and 11.67% non-Hispanic Black. No statistically significant difference in gender distribution was observed between the two groups (*p* > 0.05). However, statistically significant differences were found in age, race, education level, marital status, household income, BMI, hypertension, hyperlipidemia, diabetes, smoking history, and alcohol consumption history (*p* < 0.05).

**Table 1:** Baseline characteristics of the study population by nocturia.

Variable	Overall (n = 2099)	Without nocturia (n = 1,408)	With nocturia (n = 691)	*p* value
Age,(years)	40.875 ± 11.386	39.628 ± 11.260	43.415 ± 11.224	<0.001
Gender, *n* (%)				0.064
Male	938 (44.688%)	649 (46.094%)	289 (41.823%)	
Female	1,161 (55.312%)	759 (53.906%)	402 (58.177%)	
Race, *n* (%)				<0.001
Mexican American	172 (8.194%)	119 (8.452%)	53 (7.670%)	
Other Hispanic	206 (9.814%)	129 (9.162%)	77 (11.143%)	
Non-Hispanic White	1,226 (58.409%)	872 (61.932%)	354 (51.230%)	
Non-Hispanic Black	245 (11.672%)	126 (8.949%)	119 (17.221%)	
Other Race – Including Multi-Racial	250 (11.910%)	162 (11.506%)	88 (12.735%)	
Marital status, *n* (%)				<0.001
Married/Living with partner	1,104 (52.596%)	782 (55.540%)	322 (46.599%)	
Widowed/Divorced/Separated	380 (18.104%)	207 (14.702%)	173 (25.036%)	
Never married	615 (29.300%)	419 (29.759%)	196 (28.365%)	
Education levels, *n* (%)				<0.001
Less than 9th grade	39 (1.858%)	19 (1.349%)	20 (2.894%)	
9-11th grade	125 (5.955%)	63 (4.474%)	62 (8.973%)	
High school graduate/GED or equivalent	366 (17.437%)	218 (15.483%)	148 (21.418%)	
Some college or AA degree	683 (32.539%)	441 (31.321%)	242 (35.022%)	
College graduate or above	886 (42.211%)	667 (47.372%)	219 (31.693%)	
Poverty ratio, *n* (%)				<0.001
<1	310 (14.769%)	161 (11.435%)	149 (21.563%)	
≥1	1789 (85.231%)	1,247 (88.565%)	542 (78.437%)	
BMI, (kg/m^2^)	29.987 ± 7.694	29.175 ± 7.266	31.643 ± 8.263	<0.001
Smoke, *n* (%)				<0.001
No	1,290 (61.458%)	916 (65.057%)	374 (54.124%)	
Yes	809 (38.542%)	492 (34.943%)	317 (45.876%)	
Alcohol user, *n* (%)				<0.001
No	804 (38.304%)	916 (65.057%)	374 (54.124%)	
Yes	1,295 (61.696%)	492 (34.943%)	317 (45.876%)	
Hypertension, *n* (%)				<0.001
No	1,630 (77.656%)	1,157 (82.173%)	473 (68.452%)	
Yes	469 (22.344%)	251 (17.827%)	218 (31.548%)	
Hyperlipidemia, *n* (%)				<0.001
No	1,532 (72.987%)	1,086 (77.131%)	446 (64.544%)	
Yes	567 (27.013%)	322 (22.869%)	245 (35.456%)	
Diabetes, *n* (%)				<0.001
No	1928 (91.853%)	1,335 (94.815%)	593 (85.818%)	
Yes	171 (8.147%)	73 (5.185%)	98 (14.182%)	
Blood lead (umol/L)	0.038 ± 0.062	0.037 ± 0.068	0.039 ± 0.047	0.526
Blood cadmium (ug/L)	0.401 ± 0.569	0.362 ± 0.476	0.481 ± 0.716	<0.001
Blood selenium (umol/L)	2.314 ± 0.291	2.325 ± 0.286	2.293 ± 0.300	0.019
Blood mercury, total (ug/L)	1.066 ± 1.707	1.106 ± 1.728	0.983 ± 1.661	0.118
Blood manganese (ug/L)	9.794 ± 3.870	9.638 ± 3.522	10.111 ± 4.483	0.008

An analysis of blood trace mineral levels in subjects with varying nocturia statuses revealed statistically significant differences in blood manganese and blood cadmium levels, which were higher in the nocturia group compared to those without nocturia. In contrast, blood selenium levels were significantly lower in the nocturia group. No significant differences were found in blood mercury and lead levels between the two groups.

### 3.2 Relationship between blood trace elements and nocturia

As shown in Table 2, the unadjusted Model 1 revealed a significant positive association between blood cadmium and manganese levels and nocturia (OR = 1.418, 95% CI: 1.207–1.665, *p* < 0.001; OR = 1.031, 95% CI: 1.008–1.055, *p* = 0.009), while a significant negative association was found between blood selenium levels and nocturia (OR = 0.680, 95% CI: 0.492–0.940, *p* = 0.019). No significant association was observed between blood lead and mercury levels and nocturia. In Model 2, after adjusting for demographic variables, the positive association between blood manganese levels and nocturia remained statistically significant (OR = 1.031, 95% CI: 1.005–1.057, p = 0.019). There was a trend towards a positive correlation between blood cadmium and nocturia, as well as a negative correlation between blood selenium and nocturia, but these differences were not statistically significant. No significant relationship was found between blood lead and mercury levels and nocturia. In Model 3, after adjusting for health status, the results again revealed a significant positive correlation between blood cadmium and manganese levels and nocturia (OR = 1.289, 95% CI: 1.084–1.533; OR = 1.029, 95% CI: 1.004–1.053). Specifically, for every 1 μg/L increase in blood cadmium and manganese levels, the risk of nocturia increased by 28.9 and 2.9%, respectively. Conversely, a significant negative correlation was found between blood selenium levels and nocturia (OR = 0.691, 95% CI: 0.495–0.963), indicating that for every 1 μmol/L increase in blood selenium levels, the risk of nocturia decreased by 30.9%. This difference was statistically significant.

**Table 2:** Association of different trace minerals with nocturia.

Exposure	Model 1 OR (95% CI)	*p* value	Model II OR (95% CI)	*p* value	Model III OR (95% CI)	*p* value
Blood lead (umol/L)	1.560 (0.380, 6.400)	0.537	0.569 (0.065, 5.005)	0.611	1.744 (0.423, 7.193)	0.442
Blood cadmium (ug/L)	1.418 (1.207, 1.665)	<0.001	1.076 (0.912, 1.270)	0.387	1.289 (1.084, 1.533)	0.004
Blood selenium (umol/L)	0.680 (0.492, 0.940)	0.019	0.832 (0.598, 1.157)	0.273	0.691 (0.495, 0.963)	0.029
Blood mercury, total (ug/L)	0.954 (0.899, 1.012)	0.121	0.965 (0.909, 1.025)	0.248	0.981 (0.926, 1.039)	0.517
Blood manganese (ug/L)	1.031 (1.008, 1.055)	0.009	1.031 (1.005, 1.057)	0.019	1.029 (1.004, 1.055)	0.025

### 3.3 Subgroup analysis and interaction test analysis

**Figure 2:**
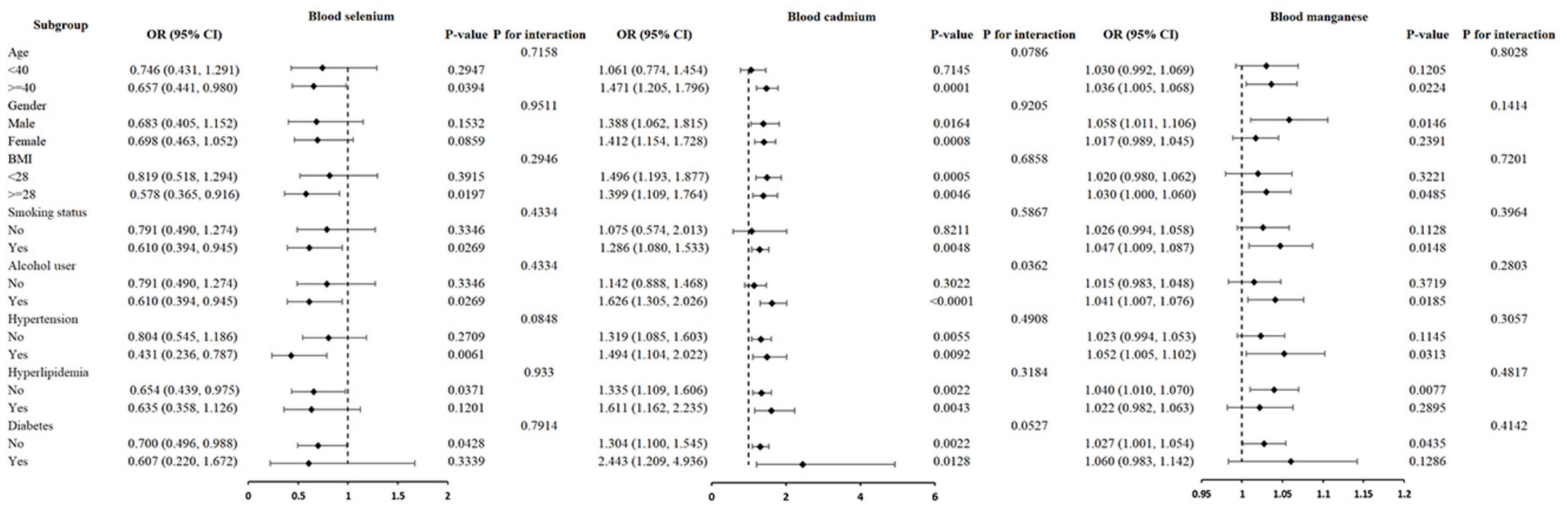
Subgroup analysis for the association between trace minerals and nocturia.

A further subgroup analysis was conducted to examine the association between blood cadmium, blood selenium, and blood manganese levels with nocturia, comparing different subgroups based on gender, age, BMI, smoking history, alcohol consumption, hypertension history, hyperlipidemia history, and diabetes history. The results of the subgroup analyses are presented in Figure 2. The negative correlation between blood selenium levels and nocturia was stronger in individuals over 40 years of age, those who were obese, smokers, alcohol consumers, hypertensive, and normolipidemic. However, this correlation was not statistically significant in other subgroups. The association between blood cadmium levels and nocturia was statistically significant across all subgroups, except for those under 40 years of age, non-smokers, and non-drinkers. Additionally, the odds ratio (OR) for blood cadmium levels with nocturia was significantly higher in the alcohol-consuming population compared to non-drinkers (OR = 1.626 vs. OR = 1.142). An interaction test revealed a significant difference in the correlation between blood cadmium levels and nocturia between alcohol consumers and non-consumers (*p* = 0.0362). The positive correlation between blood manganese levels and nocturia was more pronounced in individuals over 40 years of age, males, those with obesity, smokers, alcohol consumers, hypertensive individuals, and those with normal blood lipids and glucose levels. However, the interaction tests did not reveal any significant differences in the correlation between blood manganese levels and nocturia across the subgroups.

### 3.4 Dose–response relationship analysis of blood manganese, blood cadmium, and blood selenium levels with nocturia

**Figure 3:**
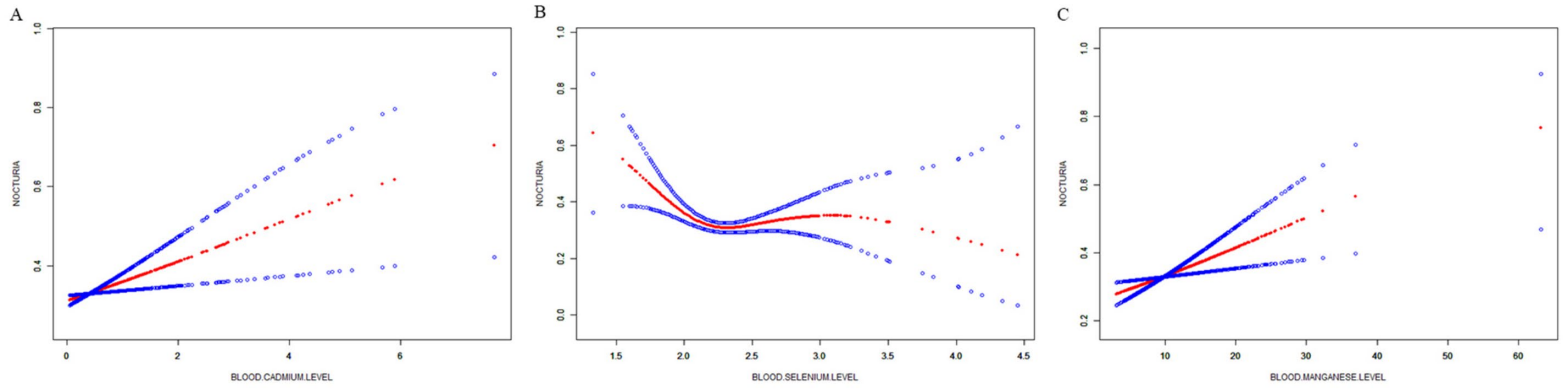
The association between trace minerals and nocturia. **(A)** Blood cadmium level and nocturia; **(B)** Blood selenium level and nocturia; **(C)** Blood manganese level and nocturia. The solid red line represents the smooth curve fit between variables. Blue bands represent the 95% confidence interval from the fit. The area between the blue lines or the blue area represents the upper and lower limits of the 95% confidence interval, and the red line represents the estimated value.

After adjusting for age, sex, and race, the dose–response relationship between blood lead, cadmium, and selenium levels and nocturia was analyzed in different subgroups using a smooth curve fit. The adjusted curve fitting revealed a nonlinear relationship between blood selenium levels and nocturia, which followed an inverted U-shaped curve (Figure 3B). The turning point, calculated using a segmented linear regression model, was determined to be 2.15 μmol/L (Table 3). A negative correlation was observed between blood selenium levels and the risk of nocturia, with a statistically significant inverse relationship (OR = 0.093, 95% CI: 0.032–0.269, *p* < 0.0002). Specifically, when blood selenium levels were <2.15 μmol/L, the risk of nocturia decreased by 90.7% for each 1 μmol/L increase in blood selenium. However, when blood selenium levels were >2.15 μmol/L, a positive correlation between blood selenium levels and the risk of nocturia was observed, though it was not statistically significant (OR = 1.132, 95% CI: 0.760–1.687, *p* = 0.541).

**Table 3:** Threshold effect analysis of Blood selenium on nocturia using a linear regression model.

Variable	Adjusted OR (95% CI)	*p* value
Inflection point	2.15	
<2.15	0.093 (0.032, 0.269)	<0.001
≥2.15	1.132 (0.760, 1.687)	0.541
Log likelihood ratio	<0.001	

**Figure 4:**
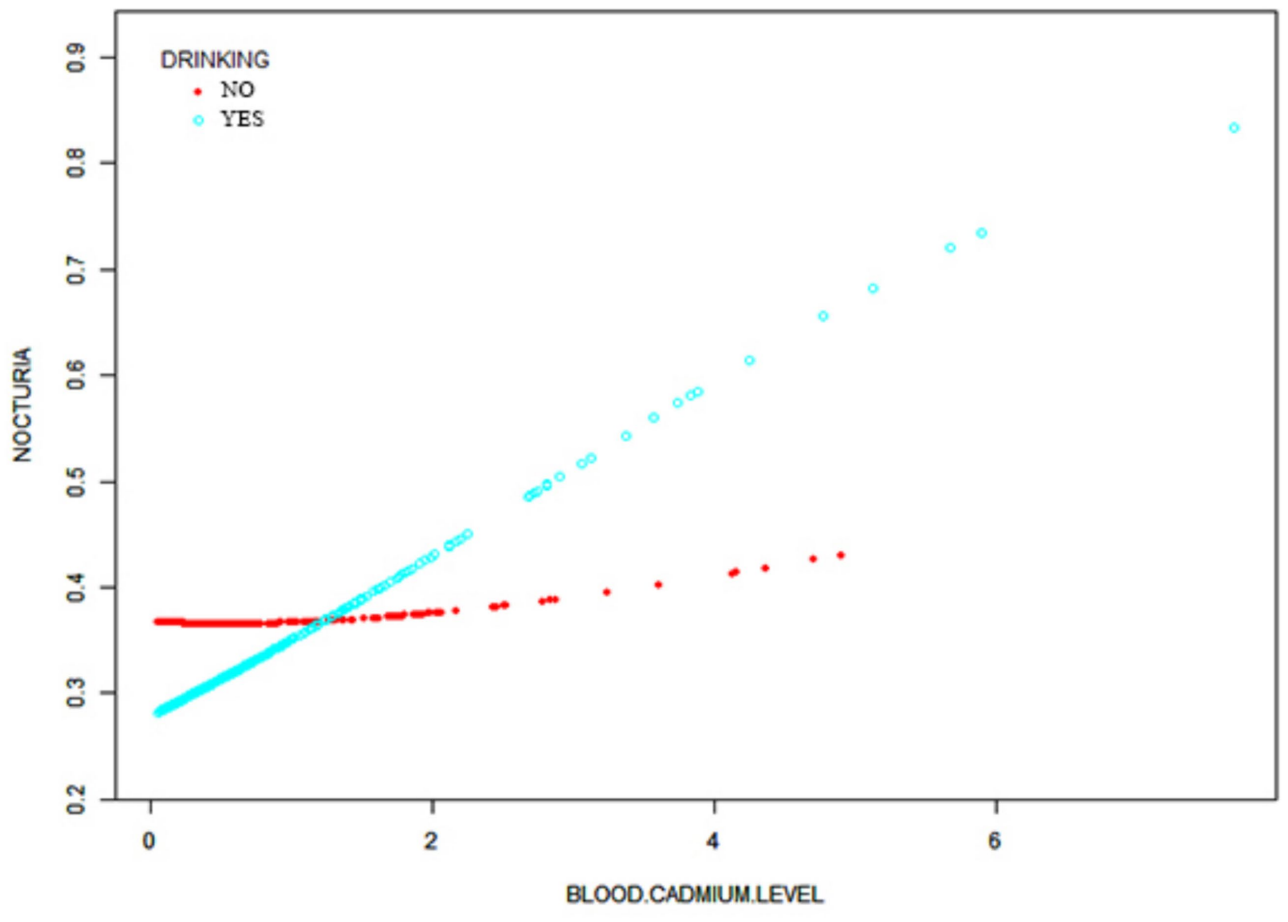
The association between blood cadmium level and nocturia stratified by drinking status.

The curve-fitting analysis of blood manganese and cadmium levels in relation to nocturia risk indicated a linear relationship (Figures 3A,C). The dose–response relationship between blood cadmium levels and nocturia in the subgroup with a history of alcohol consumption was also linear, as shown in Figure 4.

## 4 Discussion

Previously, lower urinary tract obstruction caused by benign prostatic hyperplasia (BPH) was considered the primary cause of increased nocturia in the elderly population (14, 15). However, an increasing proportion of younger individuals now suffer from nocturia, likely due to escalating environmental pollution. To explore the relationship between nocturia and blood trace mineral levels, we conducted a cross-sectional study. The results of both univariate and multivariate linear regression analyses demonstrated that elevated blood levels of cadmium and manganese were associated with an increased risk of nocturia in individuals under 60. Furthermore, an inverted U-shaped relationship was observed between blood selenium levels and nocturia, with an inflection point at 2.15 μmol/L. Lower blood selenium levels were identified as an independent protective factor against nocturia, with the protective effect being most prominent below this inflection point.

Trace minerals play a vital role in human health. However, increasing environmental pollution has led to excessive toxic heavy metals entering the food chain through air, water, and soil contamination. These metals accumulate in both animals and plants, ultimately entering the human body and posing a significant health risk (16–18). Selenium and manganese are essential minerals primarily obtained from food, water, and air (19–21). Locally grown crops in selenium-deficient soils can contribute to inadequate dietary selenium intake in humans. In contrast, lead, cadmium, and mercury are considered harmful trace minerals linked to several adverse health effects, including neurotoxicity, carcinogenicity, endocrine disruption, and reproductive and developmental toxicity. Lead exposure primarily arises from industrial activities such as informal battery recycling, electronic waste processing, metal mining, and the use of lead-based paint (22–24). Cadmium, a well-known environmental pollutant, primarily originates from agricultural and industrial activities. It has a long half-life of approximately 25–30 years and tends to accumulate in plants and animals (25, 26). A limited number of studies have explored the relationship between blood trace mineral levels and urination. In one study evaluating the association between serum micronutrients and lower urinary tract symptoms (LUTS) in older adults, it was found that serum selenium concentrations were lower in individuals with LUTS (27). A recent analysis of the NHANES database by Gao et al. identified a link between high blood cadmium levels and an increased prevalence of overactive bladder syndrome and nocturia (28). However, the precise mechanisms by which trace minerals influence bladder function remain unclear.

One of the principal manifestations of overactive bladder (OAB) is increased nocturia (29). Chronic inflammation has been shown to induce changes in bladder function and heightened sensitivity. Biopsy results have revealed chronic inflammation in the mucosal and submucosal layers of the bladder in up to 60% of patients diagnosed with OAB (30). Additionally, elevated levels of cytokines, such as monocyte chemotactic protein-1 (MCP-1), IL-5, and epidermal growth factor (EGF), have been detected in the urine of OAB patients, indicating the presence of neutrophils, eosinophils, and mast cells in the bladder (31). Selenium has been shown to modulate excessive immune responses in chronic inflammation (11, 32). At the cellular level, selenium influences various leukocyte functions, including cell adhesion, migration, phagocytosis, and cytokine secretion (33). At the molecular level, the glutathione peroxidase system in selenoproteins utilizes selenium in its active site to detoxify reactive oxygen species (ROS), such as hydrogen peroxide and phospholipid hydroperoxides (11). Selenium also plays a regulatory role in the activity of transcription factors, including nuclear factor-κB and activator protein-1, as well as the expression of related genes (34). Furthermore, selenium has been shown to decrease the levels of tumor necrosis factor-*α* and cyclooxygenase-2 produced by macrophages in response to endotoxins, while also downregulating the expression of adhesion molecules. Additionally, selenium is involved in the metabolism of arachidonic acid and eicosanoids (35). Based on these findings, it has been postulated that appropriate selenium supplementation may reduce the incidence of nocturia by improving the bladder’s inflammatory environment.

Another potential mechanism is that trace minerals may contribute to nocturia by influencing the secretion of sex hormones. It has been demonstrated that testosterone and estrogen can affect bladder function by binding to corresponding receptors in the bladder and pelvic floor muscles (36, 37), and they can directly activate BK channels, reducing the excitability of urothelial smooth muscle cells through non-genomic mechanisms (38, 39). Animal studies have shown that reduced testosterone leads to bladder dysfunction in rats (40, 41), while oestradiol supplementation reverses urinary tract epithelial damage and inflammatory cell infiltration (42). Cadmium, a toxic heavy metal, has been shown to negatively impact the testes (43). Epidemiological studies have found a negative correlation between blood cadmium levels and serum testosterone levels in adult males (44, 45). Animal studies have also confirmed that cadmium inhibits testosterone and estrogen synthesis in mouse testes (46–49). Additionally, a study by Yang et al. identified significant decreases in testosterone levels in populations exposed to manganese (50). We hypothesize that elevated blood levels of heavy metals such as cadmium may affect bladder function and contribute to increased nocturia by reducing the secretion of estrogen and testosterone. The role of trace minerals in nocturia warrants further investigation through larger studies.

To the best of our knowledge, this study is the first to report a correlation between blood trace mineral levels and nocturia in young and middle-aged adults, thus underscoring the importance of monitoring manganese, cadmium, and selenium levels in blood tests. This study has several notable strengths. Firstly, the data were derived from the NHANES database, which employed a rigorous multistage cluster sampling design. Additionally, the data underwent thorough verification by the database staff, ensuring both representativeness and reliability. Moreover, our study incorporated various covariates identified in the literature as influencing nocturia, thereby enhancing the study’s comprehensiveness. However, this study is not without its limitations. Firstly, as a cross-sectional study, it is difficult to determine the direction of the causal relationship between trace minerals and nocturia. Secondly, the study population was primarily composed of participants from the United States, which may limit the generalizability of the findings to other countries. Thirdly, despite efforts to control for confounding variables, residual confounding could still have affected the results, such as the lack of patient prostate hyperplasia-related data in the database. Furthermore, the absence of corrections for multiple subgroup analyses and interaction tests increases the risk of false positives. Therefore, future prospective studies with larger sample sizes are needed to better clarify the causal relationship between these variables and to assess the role of blood trace minerals in managing nocturia over time.

## 5 Conclusion

The results of our study revealed a significant positive correlation between blood manganese and cadmium levels and the occurrence of nocturia in young and middle-aged adults under 60. Additionally, the positive association between blood cadmium levels and nocturia was more pronounced among those who consume alcohol. A significant negative association was observed between blood selenium levels and nocturia, with evidence suggesting a threshold effect. Conversely, no statistically significant correlation was found between blood mercury and blood lead levels and nocturia. Given these findings, it may be valuable for urologists to consider the potential impact of blood trace minerals when managing nocturia in younger and middle-aged patients. However, it is important to note that the role of trace minerals in the prevention and treatment of nocturia still requires further validation through studies with larger sample sizes.

## Data Availability

The original contributions presented in the study are included in the article/supplementary material, further inquiries can be directed to the corresponding authors.
